# Formate Dehydrogenase Improves the Resistance to Formic Acid and Acetic Acid Simultaneously in *Saccharomyces cerevisiae*

**DOI:** 10.3390/ijms23063406

**Published:** 2022-03-21

**Authors:** Cong Du, Yimin Li, Ruijuan Xiang, Wenjie Yuan

**Affiliations:** 1 School of Bioengineering, Dalian University of Technology, Dalian 116024, China; 18742515846@163.com (C.D.); yiminli@dlut.edu.cn (Y.L.); xiangruijuan@bluepha.com (R.X.); 2 Ningbo Research Institute, Dalian University of Technology, Ningbo 315000, China

**Keywords:** *Saccharomyces cerevisiae*, adaptive laboratory evolution, formate dehydrogenase, formic acid, acetic acid, lignocellulose, inhibitors, cell viability

## Abstract

Bioethanol from lignocellulosic biomass is a promising and sustainable strategy to meet the energy demand and to be carbon neutral. Nevertheless, the damage of lignocellulose-derived inhibitors to microorganisms is still the main bottleneck. Developing robust strains is critical for lignocellulosic ethanol production. An evolved strain with a stronger tolerance to formate and acetate was obtained after adaptive laboratory evolution (ALE) in the formate. Transcriptional analysis was conducted to reveal the possible resistance mechanisms to weak acids, and *fdh* coding for formate dehydrogenase was selected as the target to verify whether it was related to resistance enhancement in *Saccharomyces cerevisiae* F3. Engineered *S. cerevisiae* FA with *fdh* overexpression exhibited boosted tolerance to both formate and acetate, but the resistance mechanism to formate and acetate was different. When formate exists, it breaks down by formate dehydrogenase into carbon dioxide (CO_2_) to relieve its inhibition. When there was acetate without formate, FDH1 converted CO_2_ from glucose fermentation to formate and ATP and enhanced cell viability. Together, *fdh* overexpression alone can improve the tolerance to both formate and acetate with a higher cell viability and ATP, which provides a novel strategy for robustness strain construction to produce lignocellulosic ethanol.

## 1. Introduction

The rapid reduction of fossil fuels and increased environmental pollution has prompted humans to find alternative energy sources [1]. Biofuel, a potential alternative to fossil fuels, has attracted significant interest due to its renewability and reducing carbon footprint [2]. In recent years, bioethanol produced from lignocellulosic biomass has grabbed the most attention, as carbon-neutral production promises to reduce greenhouse gas emissions [3]. Furthermore, lignocellulosic biomass is inedible, low in cost, and abundant, with 900 million tons produced every year in China, which has encouraged the Chinese government and most other countries to set a strategic goal of vigorously developing ethanol production from crop straws [4,5].

Despite the many merits of lignocellulosic feedstocks, many challenges still exist in the conversion of lignocellulosic feedstocks into fuels and chemicals by microorganisms, especially multiple toxic compounds released by pretreatment [6]. Lignocellulosic feedstocks release toxic compounds such as acetic acid, formic acid, furfural, and 5-hydroxymethylfurfural (5-HMF), which have severe inhibition on cell growth and fermentation performance at a large scale [7,8,9]. Weak acids like acetic acid and formic acid are unavoidable in lignocellulosic hydrolysates, and acetic acid has the highest concentration ranges from 1 to 15 g/L [10,11,12]. Formic acid, the other abundant weak acid released from furfural, is even more toxic than acetic acid, with its lower pKa of 3.77 and smaller size, despite the formic acid concentration being usually ten times lower than acetic acid [13,14]. Weak acids seriously damage intracellular sugar metabolism and cell growth [11,15]. A proton will be released when weak acid enters the nearly neutral yeast cytosol [16]. Accordingly, the membrane ATPase of the yeast will pump out the protons with 1 ATP per proton consumption to avoid cytoplasmic acidification, therefore additional energy would be required to excrete acetate or formate anion [17]. Moreover, weak acid anion accumulates at high concentrations in cells, which will increase the osmotic pressure of the cytoplasm, causing the increase of water inflow to restore the homeostasis and lead to cell death by intracellular pressure [18].

Consequently, numerous strategies have been proposed to reduce acetic acid and formic acid concentrations in hydrolysates [1]. For example, detoxification and minimizing inhibitor formation before fermentation [19,20]. However, detoxification strategies such as activated charcoal and washing the pretreated biomass are costly and tedious, and would lead to sugar loss, which is infeasible for large-scale industrial production [1]. Instead, ALE is applied to improve the tolerance of yeasts to inhibitors, especially acetic acid, as it is the most abundant inhibitor in the hydrolysate [8,21,22]. In its most common form, experiments involve growing cells for extended periods in a selected environment in order to select those that have acquired beneficial mutations naturally. ALE can facilitate increasingly critical mutation identification when used with high-throughput DNA sequencing and bioinformatics tools. However, formic acid and mixed inhibitors resistance are rarely investigated. So far, the achievement of resistance modification has not been satisfied as lignocellulosic hydrolysates are complex and have different resistance mechanisms [23]. Therefore, it is necessary to understand the genetic regulatory network of yeast resistance to mixed inhibitors for developing strains with a stronger tolerance to lignocellulosic hydrolysates [10].

Adaptive laboratory evolution has been proven to be the most effective way to obtain high tolerant strains, especially in lignocellulosic feedstock fermentation [24]. A formic acid-tolerant strain *S. cerevisiae* F3 was obtained after ALE. Interestingly, the evolved strain also showed enhanced tolerance to acetic acid. There is limited information about simultaneously improving the resistance of yeast to formic acid and acetic acid. Transcriptional analysis and specific enzyme activity of the evolved strains versus the original one revealed that FDH1 (GenBank accession no. NC_001147.6) might be responsible for the improved phenotype, which was confirmed by subsequent reverse genetic engineering. The corresponding resistance mechanism of recombinant *S. cerevisiae* to formic acid and acetic acid turned out to be the bidirectional catalytic activity of formate dehydrogenase. This study promotes an understanding of the stress tolerance of *S. cerevisiae* in response to weak acids, and the *fdh* gene was found to be helpful in constructing robust strains by genetic engineering. 

## 2. Results and Discussion

### 2.1. ALE and Cell Growth Test

**Figure 1:**
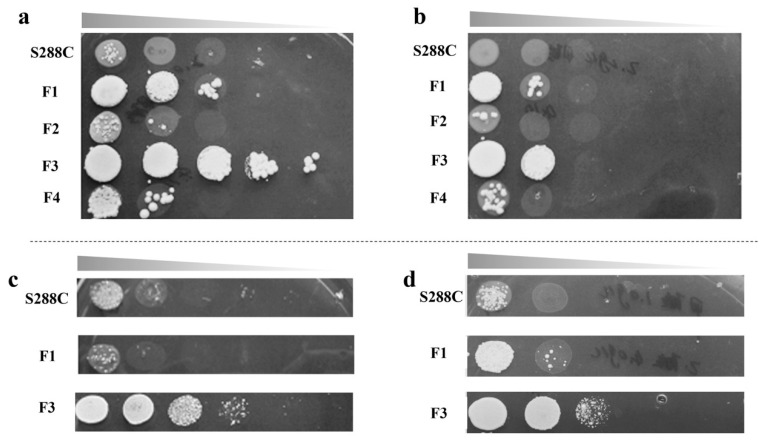
Cell growth of the *S. cerevisiae* S288C and its formic acid adapted strains F1~F4 with a 10-fold serial dilution assay in YPD medium containing (**a**) 2.0 g/L formic acid, (**b**) 2.2 g/L formic acid, (**c**) 6.0 g/L acetic acid, and (**d**) 1 g/L formic acid and 4 g/L acetic acid.

The laboratory model yeast haploid *S. cerevisiae* S288C was selected as the starting strain for ALE experiments, and was continuously cultivated in formic acid. After conducting ALE for about 100 days, four strains with improved to 2 g/L formic acid resistance were obtained from different flasks, named *S. cerevisiae* S288C F1~F4, respectively. Cell growth is shown in Figure 1a,b. 

It was seen that the growth of adaptive strains was significantly better than that of the starting strain. Among the four adaptive strains, *S. cerevisiae* S288C F1 and F3 grew better, especially the F3, which was 10^4^-fold of the original one in the presence of 2 g/L formic acid. This indicates that mutants F1 and F3 obtained a stronger tolerance to formic acid after ALE.

Consequently, *S. cerevisiae* S288C F1 and F3 were selected for the acetic acid and mixed acid resistance tests. When 6 g/L acetic acid and 1 g/L formic acid/4 g/L acetic acid mixtures were added to the YPD agar plates, respectively, F3 still had the best growth, which was about 10^3^-fold of the original one, as shown in Figure 1c,d. It is interesting that the strains adapted in formic acid also had a stronger tolerance to acetic acid or formic acid/acetic acid mixtures. There was a correlation between formic acid resistance and acetic acid resistance. 

ALE has been used for years as a practical approach to broaden the range of carbon source utilization and it improves strain tolerance to lignocellulosic-derived inhibitors [25]. *Scheffersomyces stipitis* was successfully adapted to 60% (*v*/*v*) lignocellulosic hydrolysate after culturing for 382 generations [26]. *Saccharomyces cerevisiae* strain D5A^+^ was cultured for 100 generations at a dilution rate of 0.10 h^−1^ in 60% non-detoxified hydrolysate [27], and the mutant was more robust than the parental strain in the presence of acetic acid, furfural, and HMF. Consistent with previous studies, the mutant strain F3 by ALE in formic acid was resistant to the formic acid and acetic acid, approximate to the concentration in the lignocellulosic hydrolysate. This indicated again that ALE was an effective method to get a robust strain with lignocellulosic-derived inhibitor tolerance, especially in the presence of formic acid.

### 2.2. Glucose Fermentation in the Presence of Inhibitors

**Figure 2:**
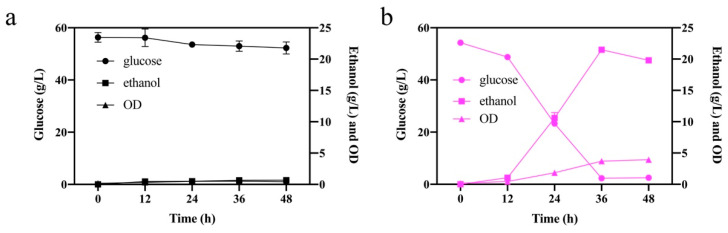
Fermentation performance of adaptive strains in flasks containing 1.8 g/L formic acid: (**a**) *S. cerevisiae* S288C and (**b**) *S. cerevisiae* F3.

We further evaluated the fermentation performance of the mutant F3 in the presence of acetic acid and formic acid, respectively. The fermentation performance of the ALE mutant F3 and the original one containing 1.8 g/L formic acid is shown in Figure 2. *S. cerevisiae* S288C (Figure 2a) barely grew, and the glucose was almost not consumed due to the severe toxicity of formic acid. The fermentation performance of F3 was better than that of the original one, consumed glucose at a faster rate with almost no residual sugar at 36 h, and the ethanol production was 21.16 ± 0.37 g/L with an ethanol yield of 0.41 ± 0.02 g/g. The above results suggest that the mutant F3 had a stronger resistance to formic acid than the starting strain.

**Figure 3:**
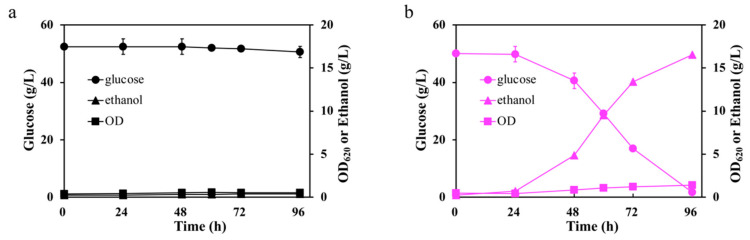
Glucose fermentation performance of *S. cerevisiae* S288C and *S. cerevisiae* F3 with 1.0 g/L formic acid and 4.0 g/L acetic acid. *S. cerevisiae* S288C (**a**) in black, *S. cerevisiae* F3 (**b**) in purple, glucose (circles), ethanol (squares), and OD_620_ (triangles).

We further evaluated the glucose fermentation of *S. cerevisiae* F3 in the presence of mixed acids (1.0 g/L formic acid and 4.0 g/L acetic acid), as shown in Figure 3. A similar phenomenon was viewed as that in 1.8 g/L formic acid. The mutant F3 exhibited a higher rate of glucose consumption, ethanol yield, and cell growth, which depleted 50 g/L glucose in 96 h with 16.5 ± 0.24 g/L ethanol production in a relatively high concentration with a yield of 0.34 ± 0.06 g/g. In contrast, the wildtype could hardly ferment glucose in harsh conditions with almost no glucose consumption. The cell growth of F3 was OD_620_ of 1.39 ± 0.04, higher than the OD_620_ of 0.52 ± 0.01 of the control. We thus summarized that the mutant F3 gained stronger tolerance to formic acid and acetic acid after ALE in formic acid.

Generally, formic acid and acetic acid are primary weak acids in lignocellulosic hydrolysates that severely damage cell growth and fermentation. However, the resistance mechanism of formic acid has been rarely investigated to our knowledge. *S. cerevisiae* GGSF16 grew slowly in the presence of 1.5 g/L formic acid and could hardly grow when the formic acid concentration increased to 1.8 g/L [28]. For *Yarrowia lipolytica*, both formic acid and acetic acid inhibited cell growth and lipid production. Formic acid and acetic acid concentrations above 1.15 g/L and 2.3 g/L, respectively, affected cell growth [29]. The mutant *S. cerevisiae* F3 adapted in formic acid had a satisfactory tolerance to 1.8 g/L and 4 g/L acetic acid. *S. cerevisiae* F3 grew very well in 1.8 g/L formic acid, and the OD_620_ increased from 0.3 to 1.39 in 96 h.

### 2.3. Transcriptional Analysis of the Evolved Strain F3

To investigate the enhanced resistance mechanism to formic acid and acetic acid of the mutant F3, a transcriptome analysis was conducted between *S. cerevisiae* F3 and the original one. 

According to the transcriptional data, clean bases of the original strain (ck) and the mutant F3 (Ad) were 2.72 G and 3.7 G, respectively. The Q20 values were 96.75% and 97.53%, and the Q30 values were 91.96% and 93.68%, respectively. There were 1528 differentially expressed genes in the total (log_2_(fold change) > 1, *q* value < 0.05), of which 726 genes were up-regulated and 802 genes were down-regulated (Figure S1).

According to the pathway enrichment analysis and differentially expressed genes, the genes in tryptophan metabolism and glycerolipid metabolism were significantly enriched, and there were 9 and 10 DEGs, respectively. Here, 24, 12, 9, and 3 DEGs were enriched in Meiosis, MAPK signaling pathway of yeast, arginine and proline metabolism, and ABC transporters, respectively (Figure S2). CTT1, DAK2, and PDR15 were the most up-regulated genes in the tryptophan metabolism, glycerolipid metabolism, and ABC transporters, respectively. Moreover, RCK1 and PMA1, which were also known to be associated with increased resistance to weak acids increased significantly. Meanwhile, FDH1, responsible for formate oxidation, was significantly up regulated. So, we chose the six genes (listed in Table 1) as the representative for RT-PCR validation, and the results of the RT-PCR were consistent with the results of RNA-seq.

The transcriptional level of RCK1 coding for a protein kinase was 7.89-fold, which proved that the overexpression of RCK1 would improve the tolerance to acetic acid by lowering the intracellular reactive oxygen species (ROS) levels in *S. cerevisiae* [30]. DAK2 was a stress responder in *S. cerevisiae*, and was upregulated in multiple stress conditions [31]. CTT1 plays a protective role in the stress condition and its overexpression was reported to prevent yeast from programmed acid damage and decreased levels of ROS [10,32]. PDR15p was strongly induced by low pH and weak acids, thus the transcript level of PDR15 was upregulated in this study [33]. PMA1 encodes the transmembrane polypeptide to pump protons out of the cell, and its overexpression promotes resistance to weak acids by enhanced proton efflux [34]. Except for FDH1, the genes described in Table 1 have been well studied in previous studies. Their overexpression could improve robustness, which might be responsible for the enhanced resistance in this study. However, the resistance mechanism of FDH1 was rarely reported. In this study, the transcription level of FDH1 in *S. cerevisiae* F3 was up regulated by 2.15-fold. Moreover, the mutant was obtained by ALE in formic acid, so we inferred that enhanced weak acid tolerance might be related to FDH1 up regulation. Moreover, formate was reported to be assimilated by FDH1 in previous reports, which protected cells from exogenous formate [18]. To prove our hypothesis, we also compared the specific activity of FDH1 in *S. cerevisiae* S288C and F3. The FDH1 activity of *S. cerevisiae* F3 (2.83 ± 0.11 g/L U/mg) was higher than that in *S. cerevisiae* S288C (1.10 ± 0.16 U/mg), as shown in Table 2. Accordingly, in this work, *fdh* was selected as the target gene to investigate its effect on formic acid and acetic acid resistance.

### 2.4. fdh Overexpression Improved Tolerance to Formic Acid

**Figure 4:**
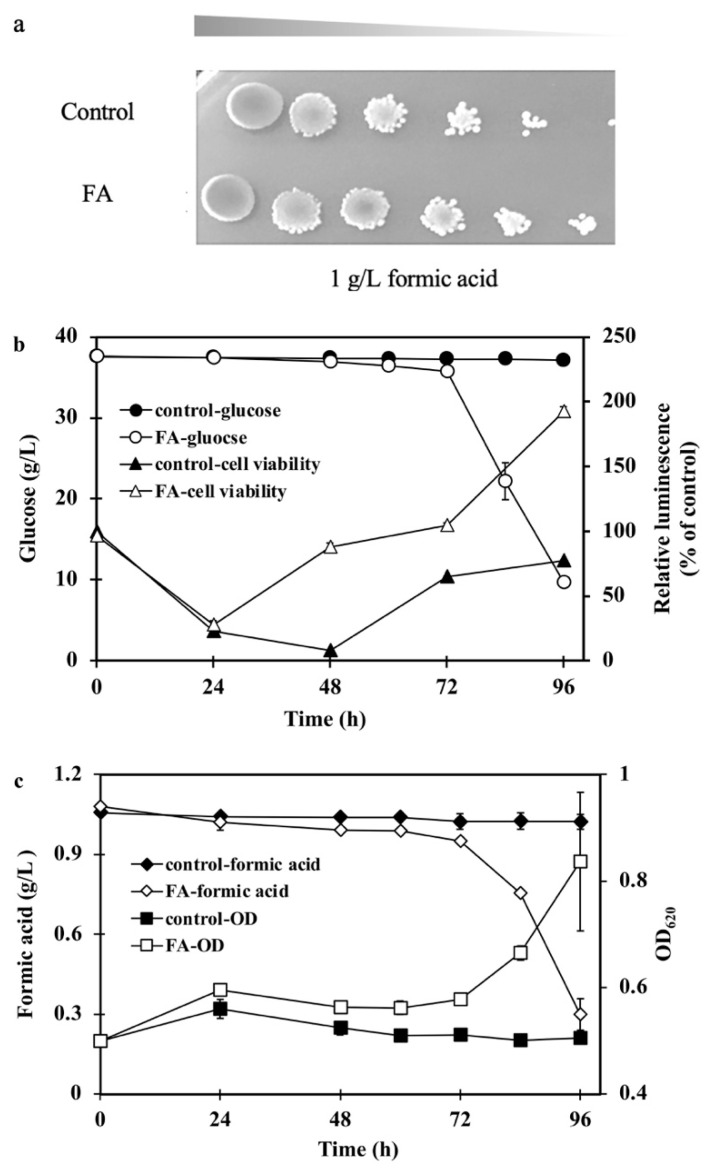
Cell growth and fermentation performance of the *S. cerevisiae* FA and the control strain: (**a**) 10-fold serial dilution assay containing 1 g/L formic acid, and (**b**,**c**) fermentation performance and cell viability assay in the presence of 1 g/L formic acid.

To verify the effect of FDH1 on the formic acid and acetic acid tolerance, endogenous *fdh* coding for formate dehydrogenase from *S. cerevisiae* F3 was amplified and overexpressed in *S. cerevisiae* S288C by ligating to multiple-copied vector pRS424-*PGK1*p to obtain *S. cerevisiae* FA (control: *S. cerevisiae* S288C with pRS424-*PGK1*p). To verify the expression level of *fdh*, the specific FDH1 activities of *S. cerevisiae* FA and the control were compared. As shown in Table 2, the FDH1 activity in *S. cerevisiae* FA increased to twice as much as the control (3.06 U/mg and 1.29 U/mg, respectively), demonstrating that *fdh* was effectively expressed in the recombinant strain *S. cerevisiae* FA. We then operated a cell growth test and glucose fermentation of *S. cerevisiae* FA and the control strain in the presence of 1 g/L formic acid to verify the effect of FDH1 on formic acid resistance. Cell growth and fermentation tests were conducted in an SD medium with *trp-* as the select marker. As the nutrient of the SD medium was less rich, and the initial pH was lower than that of the YPD medium. Experiments were conducted in 1 g/L formic acid as shown in Figure 4. 

In the cell growth test with formic acid, *S. cerevisiae* FA behaved better than the control, as shown in Figure 4a, which indicated that *fdh* overexpression could enhance formic acid tolerance. In the case of glucose fermentation in Figure 4c, the control strain could not grow without pH adjustment, while *S. cerevisiae* FA started to utilize glucose after a lag phase of 72 h with a stronger tolerance to formic acid. On the other side, when glucose started to utilize, formic acid in the medium started to reduce with only 0.3 g/L formic acid left, but there was no formic acid reduction in the control strain. In agreement with a previous report, formic acid was observed to co-utilize with glucose, and FDH1 converted formic acid to CO_2_ and NADH [18]. In the absence of oxygen, formate provided extra energy for cell anabolism by promoting NAD(P)H formation and interacting with the carbon backbone through the folate mediated C1 pathway [35]. Therefore, formic acid consumption would be attributed to the oxidation of formic acid to CO_2_ by FDH1 [23]. Furthermore, FDH1 was essential for utilizing formic acid to supply energy and power [36]. For verification, cell viability was measured during the fermentation process. The viability of the control strain at 0 h was regarded as the relative 100%, as shown in Figure 4b. After inoculation, the cell viability of both strains decreased seriously caused by the toxicity of formic acid. Cell viability started to increase for the recombinant strain FA at 24 h. At the end of the fermentation process, the cell viability of FA was about twice that of the beginning. For the control strain, the cell viability decreased from 100% to 8% in the first 48 h and then increased to 78% in the end. However, the cell viability and biomass dropped off for formic acid’s severe toxicity in the entire fermentation process. 

These results demonstrated that formic acid degradation in *S. cerevisiae* FA was caused by the formate oxidation pathway, catalyzed by FDH1. The enhanced tolerance to formic acid of *S. cerevisiae* FA would be due to formic acid’s utilization with increased cell viability.

### 2.5. fdh Overexpression Improved Tolerance to Acetic Acid

**Figure 5:**
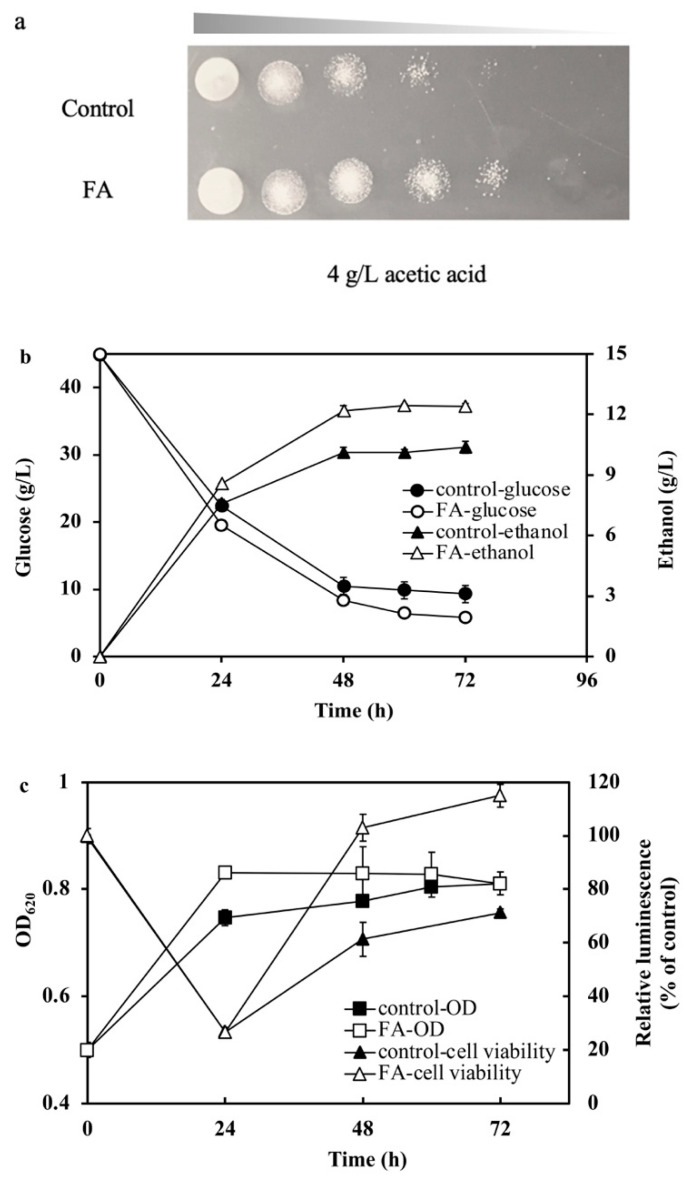
Cell growth and fermentation performance of the *S. cerevisiae* FA and the control strain: (**a**) 10-fold serial dilution assay containing 4 g/L acetic acid and (**b**,**c**) fermentation performance and cell viability assay in the presence of 4 g/L acetic acid.

To investigate if the FDH1 was related to the resistance of acetic acid, cell growth, and fermentation performance of *S. cerevisiae* FA and the control strain in 4 g/L acetic acid, as shown in Figure 5.

*S. cerevisiae* FA also grew better than the control in the presence of 4 g/L acetic acid with glucose utilized faster than the control, *S. cerevisiae* FA utilized 39.1 ± 0.46 g/L glucose and the control utilized 36 ± 0.75 g/L glucose, respectively, in 72 h (*p* < 0.05). The biomass of *S. cerevisiae* FA was increased by 12% compared with the control in 24 h, which meant *S. cerevisiae* FA had a stronger tolerance to acetic acid. Moreover, the ethanol yield of FA was 0.317 ± 0.02 g/g, which was higher than that of the control strain of 0.29 ± 0.03 g/g (*p* < 0.05) in the presence of acetic acid. This suggested that FDH1 contributed to cell growth and ethanol yield. Recently, intense efforts have been focused on resistance or utilization of acetic acid to relieve its inhibition on microbiology [7,37]. Resistance or utilization mechanisms of formic acid or mixture of formic acid/acetic acid stress were rarely done. This work proved that *fdh* overexpression simultaneously increased the resistance to formic acid and acetic acid, which was an important finding in the resistance mechanism and FDH1 functions. 

There are typically two types of FDHs: NAD^+^-dependent and NAD^+^-linked/metal-containing protein, which both can break down formic acid into CO_2_ to detoxify the inhibitory of formic acid as in equation (1) [38,39]. FDHs were also reported as a bidirectional enzyme that could convert CO_2_ to formate reversibly with NADH oxidation to NAD^+^ without any other organic chemicals [40].

HCOOH + NAD^+^ ⇄ CO_2_ +NADH + H^+^ (1)

Therefore, we assumed that the conversion of CO_2_ from glucose fermentation to formate catalyzed by FDH1 contributed to the enhanced resistance of *S. cerevisiae* FA to acetic acid, as 31 ± 1 mg/L formate was observed in the fermentation process with acetic acid addition. However, the control strain did not produce formate, as listed in Table 3. 

According to the Paris Agreement and greenhouse emissions, the conversion of CO_2_ to formic acid by the FDH1 has become a hot topic worldwide. NAD^+^-dependent FDH from *Candida boidinii* has been applied for CO_2_ reduction, resulting in 0.61 mM formate production [41]. It has also been demonstrated that NAD^+^-dependent FDH from *Thiobacillus* sp. KNK65MA has a high CO_2_ reduction activity for the production of formate, which has a 21.2-fold higher turnover number compared with FDH from *C. boidinii* [42]. NAD^+^-dependent FDH of *S. cerevisiae* has also been reported to be capable of bio-fix CO_2_ [43]. In the process of CO_2_ bio-fixation catalyzed by FDH1, NADH is needed, which is generated during glucose fermentation, where 1 mole of NADH was oxidized to NAD^+^ to produce 2 ATP as the direct energy through oxidative phosphorylation [44]. Thus, we inferred that the process of NADH oxidation by FDH1 enhanced oxidative phosphorylation to produce ATP.

To verify our hypothesis, the ATP assay via cell viability of *S. cerevisiae* FA was conducted during the glucose fermentation in the presence of 4 g/L acetic acid. Cell viability of the control strain at 0 h was regarded as the relative 100%. At the beginning of fermentation, the viability of both *S. cerevisiae* FA and the control decreased seriously after the adaption to acetic acid, and the cell viability of two strains went up at 24 h. Cell viability of *S. cerevisiae* FA increased quickly to 138%, higher than initially. Although the viability of the control started to increase after 24 h, the final viability was only 97%, which was lower than *S. cerevisiae* FA. So, we concluded that *fdh* overexpression improved acetic acid tolerance by converting NADH to generate additional ATP, which helped the yeast cell pump protons from acetic acid out in *S. cerevisiae* [45,46]. On the other hand, in a previous study, CO_2_ was proven to be an electron acceptor for NADH oxidation and ethanol productivity, consistent with our experimental results [47]. Therefore, we believe that increasing the ethanol yield in this study was due to the oxidation of NADH, which was produced from fixing CO_2_ by FDH1 overexpression.

This study confirmed that FDH1 contributed to the tolerance of formic acid and acetic acid with two different mechanisms. The resistance mechanism to formic acid of *fdh* overexpression was due to the decomposition of formic acid. However, resistance to acetic acid resulted from additional ATP generation to improve cell viability. These findings offered a new understanding of the function of FDH and provided a simple method to improve the tolerance to weak acids simultaneously.

## 3. Materials and Methods 

### 3.1. Strains and Media

The strain employed in this study was haploid *S. cerevisiae* S288C with Δ*trp* stored in our lab and the yeast culture was conducted in the YPD medium.

### 3.2. ALE in the Formic Acid

*S. cerevisiae* S288C was transferred from a single colony to the YPD medium (20 g/L glucose, 20 g/L peptone (AOBOX, 01-001), 10 g/L yeast extract (AOBOX, 01-012)) without formic acid or acetic acid, inoculated at 30 °C and 150 rpm for 24 h in shake flasks, then the cells were collected and incubated to a YPD medium with different concentrations of formic acid at an initial OD_620_ of 0.3. Cultivation was carried out at 30 °C and 150 rpm for 24–48 h, the initial concentration of formic acid was 0.2 g/L. When OD_620_ reached 2.0 (about 5–7 generations), we stopped the culture and collected the cells, and the cells were then transferred into a new medium with 0.2 g/L more formic acid. The above steps were repeated until the cell growth rate was significantly increased. After being adapted for about 100 days, the cells grew well in the final formic acid concentration of 2 g/L. The collected cells were spread on YPD plates containing inhibitors and were cultured for 24–48 h at 30 °C. A single colony with a superior growth performance was selected for the YPD medium at 30 °C and 150 rpm for 24–48 h, and was stored in 20 % glycerol at −80 °C for use.

### 3.3. Cell Growth Tests and Glucose Fermentation with Inhibitors

A single colony was selected from the YPD plate and was inoculated into a YPD liquid medium, and shake cultured at 30 °C for 24–36 h. For the inhibitor tolerance evaluation, cells of OD_620_ = 10 were serially diluted to 10^−5^ and then spotted onto YPD agar plates (20 g/L glucose, 20 g/L peptone, 10 g/L yeast extract, and 20 g/L agar) supplemented with various concentrations of formic acid and acetic acid. The yeast was cultivated at 30 °C for 48–72 h and then photographed. For the glucose fermentation with inhibitors, the yeast was collected and then transferred to the fermentation medium containing 55 g/L glucose, 20 g/L peptone, and 10 g/L yeast extract with various concentrations of inhibitors (formic acid and acetic acid) with the same initial OD_620_ of 0.3. Fermentations were conducted at 30 °C and samples were taken every 12 h for glucose, metabolites, and biomass analysis. The above experiments were replicated three times to get consistent results. The data were expressed as the mean ± SD. 

### 3.4. Transcriptional Analysis of Domesticated Strain F3

Yeast cells were cultivated in 100 mL of YPD medium for 24 h, at 30 °C and 150 rpm. Activation of the cultures, seed cultures, and the inoculation of two strains was conducted simultaneously with the same operation to ensure they were harvested at comparable states. Cell pellets were collected by centrifugation at 10,000 rpm 4 °C for 5 min, and then cells were frozen in liquid nitrogen. The total RNA of every sample was extracted using the RNeasy^®^ Mini Kit (Qiagen, Hilden, Germany) according to the manufacturer’s instructions. The 2130 Bioanalyzer (Agilent Technologies, Santa Clara, CA, USA) was used to determine the RNA quality. A total amount of 3 μg RNA per sample was used as the input material for the RNA sample preparations. Preparation of cDNA library and sequencing were performed by Illumina HiSeq 4000 and PE150 at the Novogene. Clean data (clean reads) were obtained by removing reads containing adapter, reads containing ploy-N, and low-quality reads from raw data. At the same time, using the Q20, Q30, and GC content, the clean data were calculated. Data were uploaded to NCBI (Accession number: PRJNA813024). 

Differential expression analysis was performed using the DESeq R package (1.18.0). The resulting *p*-values were adjusted using the Benjamini and Hochberg’s approach for controlling the false discovery rate. Genes with an adjusted *p* value < 0.05 (*q* value) found by DESeq were assigned as differentially expressed. KOBAS software was used to test the statistical enrichment of the differential expression genes in KEGG pathways. 

RT-PCR was conducted to further verify the gene expression levels obtained by RNA-Seq. We used the same total RNA samples for the RNA-seq reactions. According to the manufacturer’s protocol, the total RNA of each sample (1 µg) was reverse transcribed to cDNA using the PrimeScript^®^RT reagent Kit (RRO47Q, Takara Bio Inc., Dalian, China). Genes in Table 1 were selected, and the primers are listed in Table S1. cDNA was diluted 10 times as the DNA template for the qPCR reaction, and ddH2O was used as the negative control. The system and conditions were conducted as the manufacturer’s protocol of SYBR^®^ *Premix Ex Taq*™ II (Takara Bio Inc.). Finally, the actin gene (ACT1) was selected as the endogenous reference gene. Data analysis of the fold change was determined by $2^{-△△C_{T}}$.

### 3.5. fdh Overexpression in S. cerevisiae and Inhibitors Tolerance Assessment

*S. cerevisiae* S288C was used as the host in this study for overexpressing *fdh*. To increase the expression level of *fdh*. in *S. cerevisiae*, multi-copy plasmid pRS424 was applied. The coding region of the FDH1 was amplified by PCR with primers (5′-3′) attGCGGCCGCtatgtcgaagggaaaggttttg and acgcgcGTCGACttatttcttctgtccataag. The amplified products were ligated to the pRS424-*PGK1*p restricted by NotI and SalI (underlined letters). After sequencing verification by Sanger in Sangon Biotech (Shanghai, China) Co., Ltd., the plasmid was transformed into *S. cerevisiae* S288C to obtain recombinant strain *Saccharomyces cerevisiae* FA and *S. cerevisiae* S288C with pRS424 plasmid, which did not overexpress *fdh,* was used as the control.

For the inhibitor tolerance evaluation, engineered yeasts were spotted onto SD agar plates (40 g/L glucose and 6.7 g/L yeast nitrogen base without amino acids with appropriate supplements, 20 g/L agar) containing various concentrations of formic acid and acetic acid. The fermentation experiments were operated in an SD medium (40 g/L glucose and 6.7 g/L yeast nitrogen base without amino acids with appropriate supplements). Then, 4 g/L acetic acid and 1 g/L formic acid were supplemented into the medium, respectively. The experiments were repeated three times to get consistent results. The data were expressed as the mean ± SD. 

### 3.6. FDH1 Activity Assay

The FDH1 activity of strains *S. cerevisiae* S288C, F3 and *S. cerevisiae* S288C-pRS424, *S. cerevisiae* FA were determined. *S. cerevisiae* S288C, F3 and *S. cerevisiae* S288C-pRS424, *S. cerevisiae* FA were cultured for 12–24 h in YPD media and SD media, respectively, at 30 °C. The cells (OD_620_ = 1) were collected and suspended in 10 mM sodium phosphate buffer for cell disruption. The FDH1 activity was assayed in a reaction mixture (1 mL) containing 100 µL cell extract, 100 mM mercaptoethanol, 1.67 mM NAD^+^, and 167 mM sodium formate in 100 mM sodium phosphate buffer (pH = 7.5) and the production of NADH was measured at 340 nm. The total protein concentrations in the cell extract were determined by TaKaRa BCA Protein Assay Kit (code no. T9300A). One unit was defined as the amount of enzyme that produced 1 mmol of NADH per minute at 30 °C. The data were expressed as the mean ± SD. 

### 3.7. Determination of Cell Viability 

Cells of about OD_620_ = 0.5 were collected at intervals. Before cell viability detection, lyase was added to pretreat the cell for 2 h. Cell viability in the presence of acetic acid and formic acid in SD medium was assayed, followed by CellCounting-Lite 2.0 Luminescent Cell Viability Assay (Vazyme Biotech Co., Ltd., Nanjing, China). CellCounting-Lite2.0 used in this study is a cell viability detection reagent based on the luciferase system. The reagent contains high-purity luciferin and thermostable luciferase. When we added this product to the cell culture to lyse the cells and release ATP, and the reaction shown in the figure below can be produced, and a stable “light” type signal can be issued. The luminescence intensity is proportional to the amount of ATP, that is, the number of living cells within a certain range. So, we used cell viability to present relative ATP levels. Luminescence was measured by SpectraMax M2e (Molecular Device) at 560 nm. The data were expressed as mean ± SD. The bars in the figures indicate the ranges of the standard deviation.

### 3.8. Analytical Methods 

Cell growth was measured by Multiskan ascent 354 at 620 nm. The sugar and product concentrations were measured by HPLC system (Acchrom S6000) using BIO-RAD Aminex HPX-87H Column. The mobile phase was 0.01 N H_2_SO_4_ at a column temperature of 50 °C, 0.5 mL/min. Glucose and ethanol were determined by a reflective index detector, and the formic acid concentration was measured by a UV detector at 210 nm. The data were expressed as the mean ± SD. The bars in the figures indicate the ranges of the standard deviation.

## Figures and Tables

**Table 1:** Transcription levels of differential genes in *S. cerevisiae* F3 compared with S288C.

Gene	log_2_FC	Description
RCK1 [30]	7.89	Protein kinase involved in the response to oxidative stress
DAK2 [31]	4.21	Required for detoxification of dihydroxyacetone (DHA), involved in stress adaptation
CTT1 [10,32]	3.10	Has a role in protection from oxidative damage by hydrogen peroxide
PDR15 [33]	2.81	Multidrug transporter and general stress response factor implicated in cellular detoxification
FDH1 [18]	2.15	May protect cells from exogenous formate
PMA1 [34]	2.10	Pump protons out of cell, major regulator of cytoplasmic pH and plasma membrane potential

**Table 2:** Specific FDH1 activity in U/mg of total protein.

Strain	Activity (U/mg)	Medium
*S. cerevisiae* S288C	1.10 ± 0.16	YPD
*S. cerevisiae* F3	2.83 ± 0.11	YPD
Control	1.29 ± 0.09	SD
*S. cerevisiae* FA	3.06 ± 0.21	SD

Control: *S. cerevisiae* S288C with pRS424-*PGK1*p. *S. cerevisiae* FA: *S. cerevisiae* S288C with pRS424-PGK1p-fdh.

**Table 3:** Formic acid accumulation in the presence of acetic acid.

Strain	Formic Acid Concentration (mg/L)
Control	ND
*S. cerevisiae* FA	31 ± 1

ND: Not detected.

## Data Availability

Data is contained within the article or Supplementary Materials.

## Supplementary Materials

The following supporting information can be downloaded at: https://www.mdpi.com/article/10.3390/ijms23063406/s1.
