# Supplementary material for: Formate Dehydrogenase Improves the Resistance to Formic Acid and Acetic Acid Simultaneously in Saccharomyces cerevisiae
Source: Int J Mol Sci. 2022 Mar 21;23(6):3406. doi: 10.3390/ijms23063406 (PMC8954399; doi:10.3390/ijms23063406)
Supplement: Supplementary file 1 [file ijms-23-03406-s001.zip › ijms-1589546-supplementary-final.pdf]

International Journal of
Molecular Sciences

MDPI

Article

Formate dehydrogenase improves the resistance to formic acid and acetic acid simultaneously in *Saccharomyces cerevisiae*

Cong Du [1,†], Yimin Li [1,†], Ruijuan Xiang [1] and Wenjie Yuan [1,2,*]

[1] School of Bioengineering, Dalian University of Technology, Dalian 116024, China; 18742515846@163.com (C.D.); yiminli@dlut.edu.cn (Y.L.); xiangruijuan@bluepha.com (R.X.)
[2] Ningbo Research Institute, Dalian University of Technology, Ningbo 315000, China
* Correspondence: ywj@dlut.edu.cn
† These authors contributed equally to this work.

Table S1. Primers of RT-PCR.

Primer	Sequence	Product size
RCK1-F	CACGGATGACCAAGCTCCTGTTG	145
RCK1-R	CGAAACCAGTTTGTGGATGGCAAC	145
DAK2-F	AGAAGTTGCCGAAGTTGCTCAGTC	80
DAK2-R	CAGGAACCGTGCAGTGATCAAGAG	80
CTT1-F	CTGGAGATAAGGCTGCTGAACTGTC	132
CTT1-R	TTGCTTGTTCGGGTGTCATTGTTTG	132
PDR15-F	TTTCGCATACAAGGGACACCAAGG	84
PDR15-R	CCGCAATGGATACACGCTTTCTTTC	84
FDH1-F	CGGTAGACAGGGAGTTGAAAGACG	114
FDH1-R	CGGTTACACAGAGCTTCAGGTTAGG	114
PMA1-F	AAGGCTAAGGACGCTTTGACCAAG	92
PMA1-R	GGAGATTCGACAACGGCAGTAACC	92
ACT1-F	AAGTGTGATGTCGATGTCCGTAAGG	120
ACT1-R	AGATGGAGCCAAAGCGGTGATTTC	120

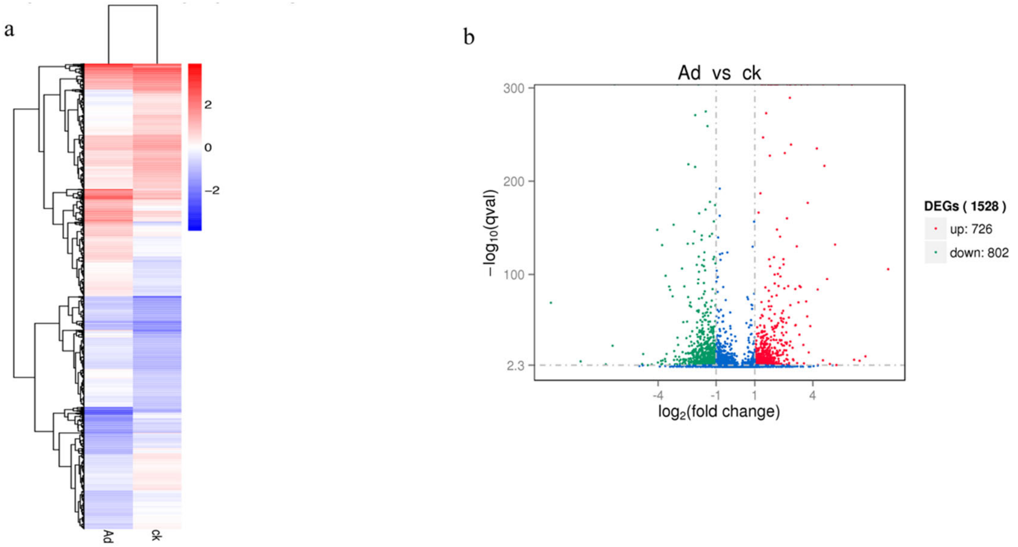

Figure S1. Clusters analysis and Volcano Plot of DEGs. (**a**) Cluster analysis of differentially expressed genes; (**b**) Volcano Plot, red plots were up-regulated, blue plots were down-regulated, blue plots were not significantly different.

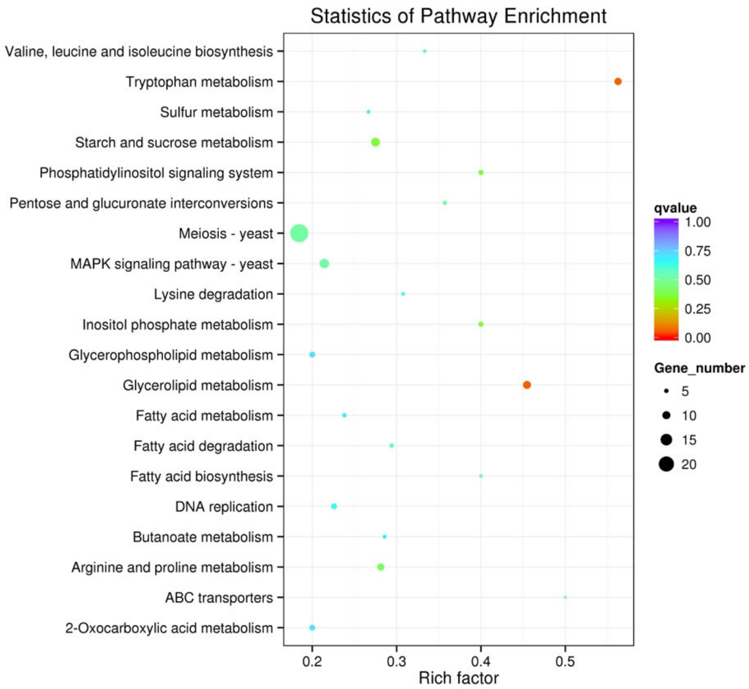

Figure S2. Statistics of pathway enrichment.
